# Supplementary material for: Group decision-making is optimal in adolescence
Source: Sci Rep. 2018 Oct 22;8:15565. doi: 10.1038/s41598-018-33557-x (PMC6197285; doi:10.1038/s41598-018-33557-x)
Supplement: Supplementary file 1 — Supplementary Information [file 41598_2018_33557_MOESM1_ESM.docx]

**Supporting Information**

Group decision-making is optimal in adolescence

Simone PW Haller^†^, Dan Bang^†^, Bahador Bahrami, Jennifer YF Lau

^†^These authors contributed equally

*Age*

**Figure S1** shows age in years for each dyad member in each age group.


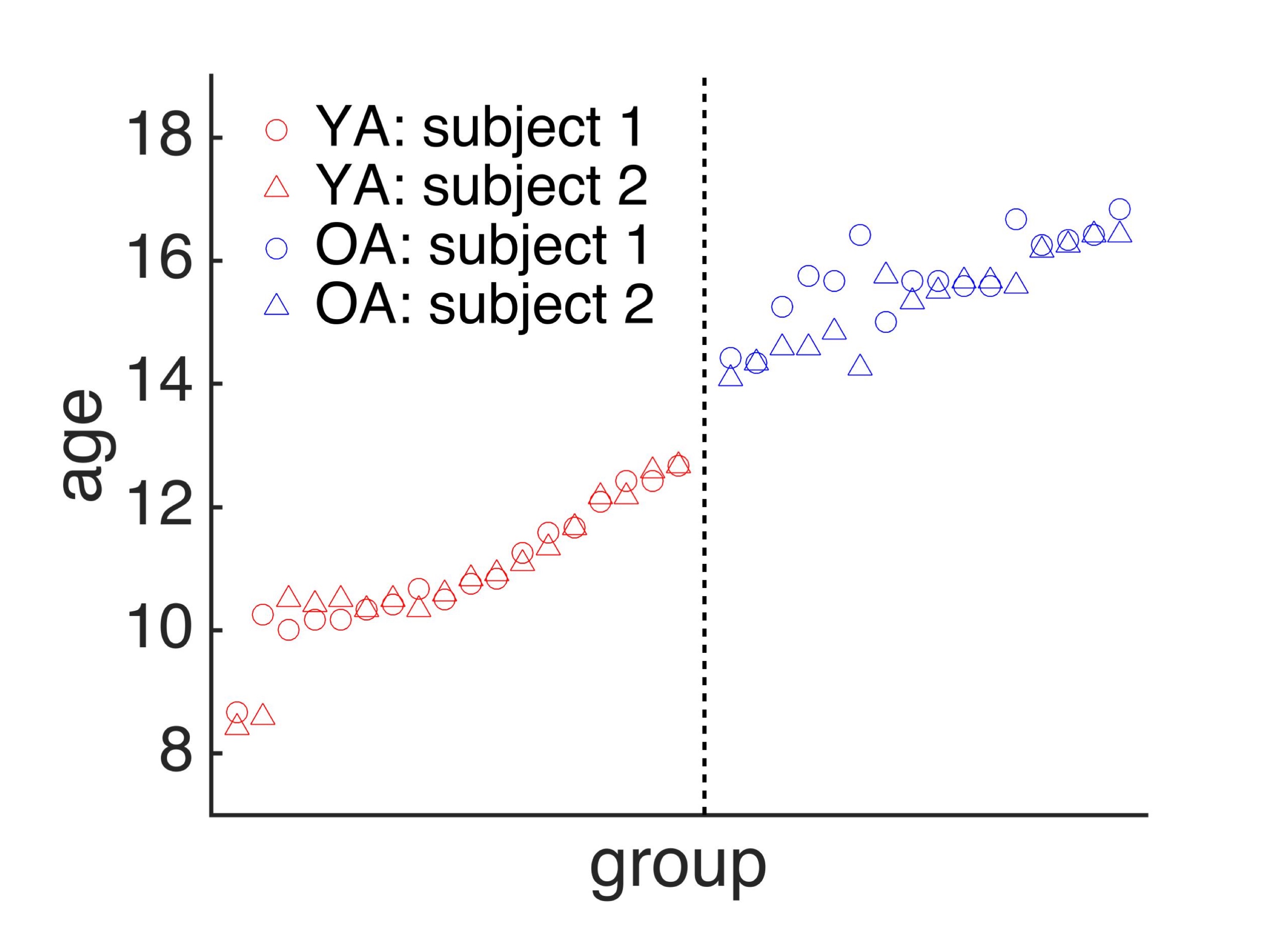


**Figure S1.** Age in years for all participants.

*Age as a continuous measure*

To further investigate developmental differences in individual and joint behaviour, we used multiple linear regression to assess the relationship between age (individual age or the mean age of a dyad) and the measures of individual and joint performance. To take into account non-linear trajectories, we included both a linear term (age) and a quadratic term (age^2^) in our regression model.

Individual performance

We observed significantly positive developmental gradients for accuracy (**Figure S2A**), sensitivity (**Figure S2B**) and choice reaction time (**Figure S2C**). We did not observe a developmental gradient for egocentric bias (**Figure S2D**); while there were individual differences in egocentric bias (dot dispersion), these did not vary with age.

Joint performance

We did not observe a developmental gradient for similarity of sensitivity (**Figure S3A**). However, we observed marginally positive developmental gradients for collective benefit (**Figure S3B**) and optimality (**Figure S3C**) together with a marginally negative developmental gradient for deliberation time (**Figure S3D**). These results are most likely driven by the temporal deterioration of joint performance among younger participants (**Figure 5**).

**
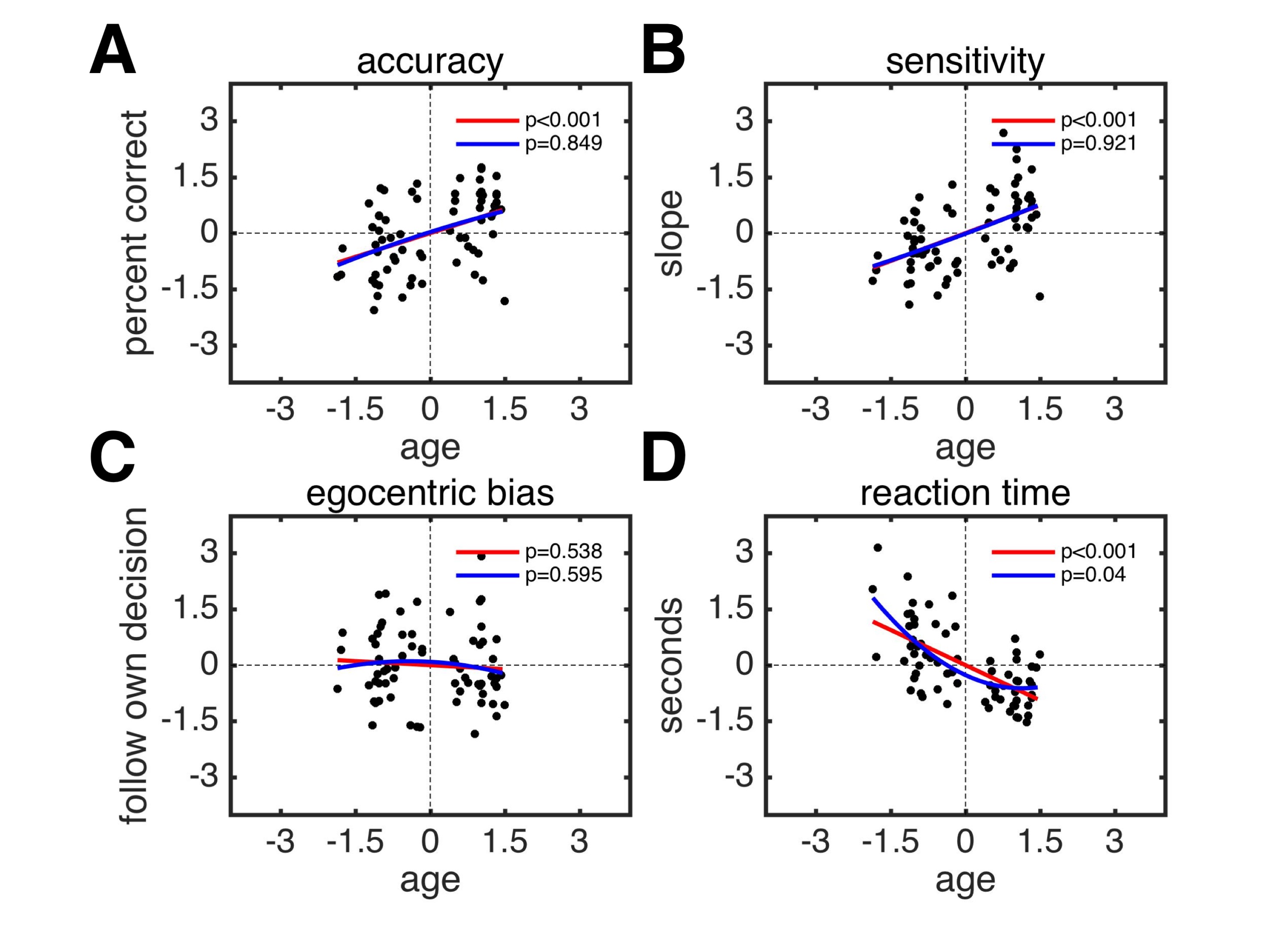
**

**Figure S2.** Developmental gradients for individual behaviour. **A**, Accuracy. **B**, Sensitivity. **C**, Egocentric bias. **D**, Reaction time. **A-D**, Each dot is a participant. The lines indicate the slopes for the linear (red) and the quadratic (blue) terms for age; the *p*-values indicate the significance of the terms. All variables were *z*-scored before being entered into the regression model.

**
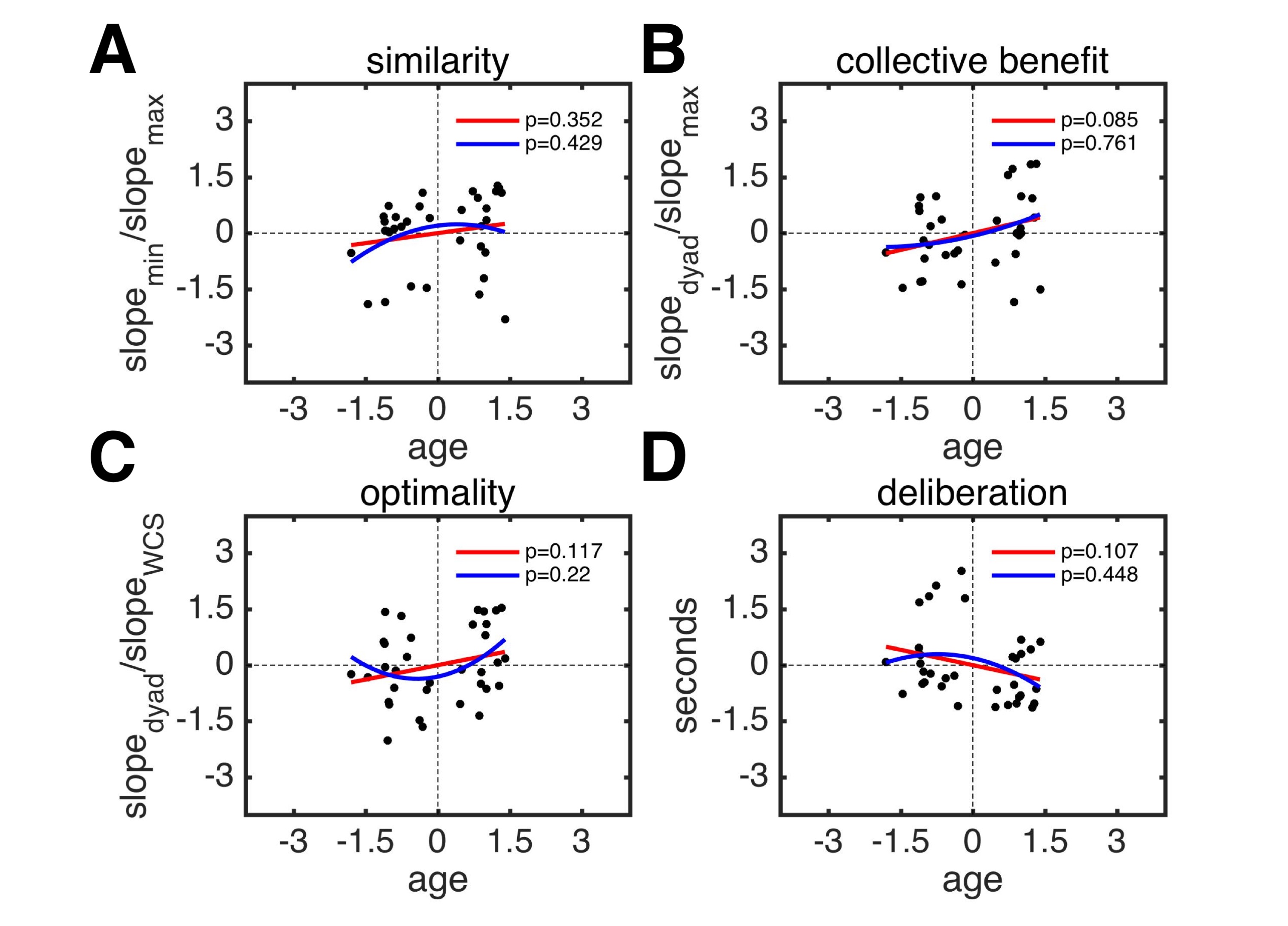
**

**Figure S3.** Developmental gradients for joint behaviour. **A**, Similarity of sensitivity. **B**, Collective benefit. **C**, Optimality. **D**, Deliberation time. **A-D**, Each dot represents a dyad. The lines indicate the slopes for the linear (red) and the quadratic (blue) terms for the mean age of dyads; the *p*-values indicate the significance of the terms. All variables were *z*-scored before being entered into the regression model.
